# Re-Sequencing Data for Refining Candidate Genes and Polymorphisms in QTL Regions Affecting Adiposity in Chicken

**DOI:** 10.1371/journal.pone.0111299

**Published:** 2014-10-21

**Authors:** Pierre-François Roux, Morgane Boutin, Colette Désert, Anis Djari, Diane Esquerré, Christophe Klopp, Sandrine Lagarrigue, Olivier Demeure

**Affiliations:** 1 INRA, UMR1348 PEGASE, Saint-Gilles, France; 2 Agrocampus Ouest, UMR1348 PEGASE, Rennes, France; 3 Université Européenne de Bretagne, Rennes, France; 4 INRA, SIGENAE, Castanet-Tolosan, France; 5 INRA, UMR1388 GenPhySE, GeT-PlaGe, Castanet-Tolosan, France; Sabanci University, Turkey

## Abstract

In this study, we propose an approach aiming at fine-mapping adiposity QTL in chicken, integrating whole genome re-sequencing data. First, two QTL regions for adiposity were identified by performing a classical linkage analysis on 1362 offspring in 11 sire families obtained by crossing two meat-type chicken lines divergently selected for abdominal fat weight. Those regions, located on chromosome 7 and 19, contained a total of 77 and 84 genes, respectively. Then, SNPs and indels in these regions were identified by re-sequencing sires. Considering issues related to polymorphism annotations for regulatory regions, we focused on the 120 and 104 polymorphisms having an impact on protein sequence, and located in coding regions of 35 and 42 genes situated in the two QTL regions. Subsequently, a filter was applied on SNPs considering their potential impact on the protein function based on conservation criteria. For the two regions, we identified 42 and 34 functional polymorphisms carried by 18 and 24 genes, and likely to deeply impact protein, including 3 coding indels and 4 nonsense SNPs. Finally, using gene functional annotation, a short list of 17 and 4 polymorphisms in 6 and 4 functional genes has been defined. Even if we cannot exclude that the causal polymorphisms may be located in regulatory regions, this strategy gives a complete overview of the candidate polymorphisms in coding regions and prioritize them on conservation- and functional-based arguments.

## Introduction

Over the past decade, a lot of studies that aimed at dissecting the genetics of complex traits have been carried out, focused on identifying causal genes and polymorphisms for disease phenotypes or traits of economic interest, on humans, animal models and livestock species [1], .

For such studies, genome-wide association study (GWAS), using high densities of genetic markers, based on linkage disequilibrium (LD) analyses, and permitting the identification of short length QTL regions is now commonly used. However, as technologies (high density SNP arrays) allowing LD approaches were only recently available, most of the published studies on complex traits in livestock species were based on linkage analysis (LA) approaches (as described in QTLdb [1]), and therefore described larger QTL regions.

Such large regions highlighted as impacting a complex trait using LA contain dozens of genes. Therefore, in general, only genes already known for having a link with the traits of interest are studied, while most of the genes are not even considered, as they have no functional characterization. Even doing so, for many traits the number of potential candidate genes is high and studying them one by one is time consuming. This probably explains why, while thousands of QTL were detected, only very few causal polymorphisms were identified [3]–[6]. For a few years, with the advent of next-generation sequencing (NGS), and the highly decreased whole-genome sequencing costs associated, it is now possible to sequence the whole genome of a few individuals and to access without *a priori* to all polymorphisms from key individuals, which is critical to identify causal polymorphism underlying QTL regions [7], [8]. The aim of this study was to combine QTL and NGS information to characterize regions affecting adiposity in chicken. This led to the identification of 216 missense SNPs, 5 nonsense SNPs and 3 coding indels occurring in 77 genes that underlay two QTLs. Using conservation- and functionality-based filters aiming at prioritizing polymorphisms, this number was reduced to 76 functional polymorphisms in 41 genes including 21 functional polymorphisms in 10 genes related to energetic metabolism.

## Methods

### Experimental design

A F_2_ design of 561 offspring in 5 F_1_ sire families [9] was created by inter-crossing two experimental meat-type chicken lines, the lean line and the fat line, that were divergently selected on abdominal fatness [10]. 801 backcross animals in 6 sire families derived from the F_2_ design were also used.

Broilers were fed *ad libitum* using conventional starter diet from 0 to 3 week and grower diet from 4 to 9 week. At nine weeks of age, blood was collected from all animals of the F_2_ and BC designs before slaughter. Body weight and abdominal fat weight were measured for each F_2_ and BC animal. The experimental unit where birds were kept is registered by the French Ministry of Agriculture with the license number B-37-175-1 for animal experimentation. Except blood collection, no manipulation was performed before slaughtering. Slaughtering and blood collection were performed in accordance with guideline of ethics committee in Animal Experimentation of Val de Loire that approved this study.

### Genetic markers

The F1 sires were genotyped for a set of 9126 SNPs covering the available genome (assembly 2.1 WASHUC2). A subset of 1536 SNPs was selected using MarkerSet [11], based on marker location and heterozigosity in the F_1_ population to maximize both genome coverage and marker informativity. The average density was one SNP each 0.66 cM, *i.e.* one SNP for 3 Mb. The 1362 offspring were then genotyped for those 1536 SNPs, at the National Genotyping Center (CNG, Evry, France) using Illumina GoldenGate technology (Illumina, San Diego, CA, USA). MendelSoft [12] was used to correct data for Mendelian inconsistencies. Out of the 1536 markers, 191 were eliminated due to technical or inconsistence issues (call rate lower than 85% and/or Mendelian errors higher than 5%). The chicken linkage consensus map build by Groenen *et al*. [13] was used to determine the genetic location of markers. Location of markers unavailable in the consensus map was extrapolated based on flanking markers.

### QTL mapping

QTL interval mapping was performed using QTLMap software [14]. A mixture of half and full-sib families was considered as pedigree structure, and only sire meioses were studied. For abdominal fat (AF) QTL interval mapping, sex (2 levels) and hatch group (5 levels) were used as fixed effects, while body weight at nine weeks (BW9) was used as co-variable to adjust data. A likelihood ratio test (LRT) was performed at each cM to compare the fit of two models (*i.e.* the model with a QTL at the location considered *vs.* the model without fitting any QTL effect). Chromosome-wide significance thresholds were evaluated through empirical calculations obtained by simulations under the null hypothesis. A total of 10,000 simulations was performed for each trait × chromosome combination and maximum LRT quantiles were calculated according to Harrel and Davis method [15]. Confidence intervals on QTL positions were estimated by the drop-off method [16]. Similarly to the reduction of one logarithm of odds (LOD) when using LOD scores, the maximum LRT value was reduced by 3.84 (a χ^2^ distribution with one degree of freedom for *p*<0.05) to determine a threshold. Region boundaries were then defined by the LRT locations crossing this threshold upstream and downstream of the LRT peak. The substitution effect of QTL was estimated in each sire family at the position of the LRT maximum and the significance was evaluated using a Student *t-*test.

### Whole genome re-sequencing

DNA-seq libraries from 8 sire samples were prepared using the TruSeq DNA Sample Preparation Kit (Illumina, San Diego, CA) according to the manufacturer's instructions. Briefly, paired-end libraries with a 250-bp insert size were generated using the Illumina TruSeq DNA Sample Prep Kit. The libraries were quantified using QPCR Library Quantification Kit (Agilent), controlled on a High Sensitivity DNA Chip (Agilent) and sequenced in paired-end 2×100 bp on Illumina HiSeq 2000 with TruSeq v3 Kit. Sequencing produced an average of 92% of uniquely mapped reads, *i.e.* 20.4 Gb, which stands fort a sequencing depth of 19.7 X.

### DNA-seq data preprocessing, variant and genotype callings

The read sets obtained by sequencing whole genome were aligned against the *Gallus gallus* WASHUC2.1 reference genome from Ensembl 58 using BWA v0.7.0 [17]. All alignment bam files have been indexed and filtered. PCR duplicates were removed using SAMtools rmdup. Only reads with a unique mapping hit and a phred mapping quality score greater than 30 were kept. All these steps were performed using SAMtools v0.1.19 [18]. Genome Analysis ToolKit v2.4.9 (GATK) [19] was then used for base quality score re-calibration, indel re-alignment, and variant calling with UnifiedGenotyper by using default parameters, as suggested in GATK Best Practices recommendations [19], [20]. Standard hard filtering parameters were finally used on SNPs and indels sets, according to GATK Best Practices recommendations [19], [20]. Briefly, we filtered out SNPs characterized by: QD<2.0 (quality by depth for non reference samples), MQ<40 (mapping quality across all samples), FS>60.0 (phred-scaled *p*-value of Fisher's exact test for strand bias), MQRankSum <−12.5 (mapping quality rank sum test) and ReadPosRankSum <−8 (read position rank sum test for the distance from the end of the read for reads with alternate allele); and we filtered out indels characterized by: QD<2.0, ReadPosRankSum <−20.0, InbreedingCoeff <−0.8 and FS<200.0. Finally, we removed genotypes for which the global depth DP (depth) was under 3 or higher than 60 (mean depth + 6σ), using VCFtools [21].

### Polymorphism and gene annotations

To identify positional candidate genes in highlighted QTL regions, AnnotQTL software [22] was used, providing the location of genes in a specific region using NCBI and Ensembl databases, and filtering them onto ontology annotation criterion. Thus, genes belonging to “lipid metabolic process” Gene Ontology (GO) class, and to “diabetes mellitus” and “obesity” Online Mendelian Inheritance in Men (OMIM) classes were considered as functional candidate genes.

Variant annotation was performed using a two steps procedure. First, we used Ensembl Variant Effect Predictor (VEP) [23] for a global annotation on WASHUC2.1, allowing us to focus on SNPs associated to “missense”, “stop gained” or “stop lost” coding consequences, and on indels associated to “inframe insertion”, “inframe deletion” or “frameshift” coding consequences. After a validation of these annotations with VEP on the latest reference genome Galgal4, these variants were finely analyzed using the NGS-SNP tool [24] allowing to add meta-information about conservation between orthologous sequences. For this second step, we considered conservation information between *Gallus gallus*, *Canis lupus familiaris*, *Bos taurus*, *Mus musculus*, *Rattus norvegicus*, *Sus scrofa* and *Homo sapiens*. We considered two types of conservation score provided by NGS-SNP. The first one defined by Grant *et al.* [24], termed “alignment score change” or *a* score, and based on the log-odds scoring matrix BLOSUM62, allows to range the similarity between the variant and the reference amino acid resulting from a coding variant, and amino acids in orthologous sequences. Considering the absolute value for this score, largest scores indicate that highly conserved amino acid residues are impacted and that substitution involved are less likely according to BLOSUM62 matrix. After studying how alignment score change was distributed for SNPs located on the two QTL regions, we considered as functional SNPs those having a |*a*| score higher than the first quartile value of the distribution. The second score we used was provided by the Sorting Tolerant From Intolerant (SIFT) algorithm [25], based on the principle of protein evolution and using sequence homology based approach to classify amino acid substitutions. This latter approach is based on the hypothesis that highly conserved positions tend to be intolerant to substitutions, while those with a low degree of conservation tolerate most substitutions. We considered all SNPs classified as “deleterious” by SIFT as functional (excepted for “stop gained” SNPs, for which SIFT annotation is not available). Regarding indels, as they impact sequence in a much more complex pattern than SNPs, those annotations are not available.

### Polymorphism validations by Sanger sequencing

Polymorphism validation was performed by Sanger sequencing for 8 coding SNPs and 3 coding indels. Targeted sequences were first PCR-amplified using 50 ng DNA with a Taq Uptitherm kit (Interchim). Amplicons were then purified and sent to GATC-Biotech (Konstanz, Germany) for Sanger sequencing using primers described in Table S3. Sanger traces related to indels were analyzed using Mixed Sequence Reader [26].

## Results and Discussion

### QTL analysis revealed two regions involved in the regulation of abdominal fatness

**Figure 1:**
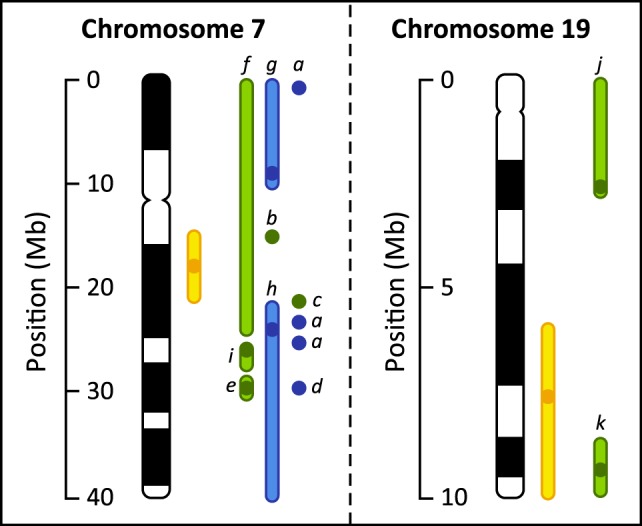
Chromosomal location of present and previously published QTLs related to abdominal fat weight. Empty boxes encompass the confidence interval of the QTL, when available. Plain boxes point out the QTL peak location, when available. QTLs colored in red are genome-wide significant (*p*<0.05), while those colored in blue are suggestive QTLs (*p*<0.2). QTLs described in the present study are colored in orange. ^a^ Ankra-Badu *et al*. [30], ^b^ Zhou *et al.* [54], ^c^ McElroy *et al.* [55], ^d^ Jennen *et al.* [56], ^e^ Tatsuda *et al.* [57], ^f^ Ikeobi *et al.* [28], ^g^ Lagarrigue *et al.* [27], ^h^ Park *et al.* [58], ^i^ Wang *et al.* [59], ^j^ Nadaf *et al.* [60], ^k^ Demeure *et al.* [9], ^l^ Tian *et al.* [61].

Whole genome QTL analysis for the abdominal fatness trait on 1362 offspring in 11 sire families using 1536 markers led to the identification of two QTLs mapped on GGA7 (*p*<0.05) and GGA19 (*p*<0.01) (Table 1). QTL effects were estimated at 0.54 and 0.45 phenotypic standard deviations and confidence interval at 10.2 cM (i.e. 6.01 Mb) and 5.7 cM (i.e. 2.91 Mb) for QTL on GGA7 and GGA19, respectively. These two regions were previously described as affecting AF ([27]–[29] for GGA7, [30] for GGA19, Figure 1), which reinforce the interest of focusing on them.

**Table 1:** QTL analysis results.

**Chromosome**	GGA7	GGA19
**QTL location (cM)**	58	52
**Size (cM)**	10.2	5.7
**Size (Mb)**	6.01	2.91
**LRT**	31.9	31.5
**Significance level^1^**	*	**
**QTL effect^2^**	0.54	0.45
**Flanking marker −**	rs15853071	rs15850508
**Flanking marker +**	rs14615490	rs13576125

1: * 5%; ** 1%; Chromosome wide significance.

2: Substitution effect, expressed in phenotypic standard deviation.

### NGS data allowed the identification of functional polymorphisms in coding regions within QTL regions

As in all QTL fine-mapping studies, we first characterized genes located in each QTL region. Using AnnotQTL [22], based on both NCBI and Ensembl databases, all genes located in both QTL regions were listed. This led to the identification of 77 genes and 84 positional candidate genes located on GGA7 and GGA19 QTL regions, respectively.

Using whole genome DNA sequencing data, we then characterized polymorphisms in both regions and revealed 39,781 and 19,755 SNPs and 4,613 and 1,829 indels (Table 2). As it is difficult to annotate and determine the impact of polymorphisms on regulatory regions – an issue that is still important in model species due to the lack of annotation in non-coding regions [31]–[33] -, only polymorphisms having an impact on coding sequences were further considered.

**Table 2:** Selection of candidate polymorphisms.

		In QTL region	And affecting protein sequence	And potentially functional
GGA7	Number of SNPs	39781	119	41
	Number of indels	4613	1	1
	Number of genes	77	35	18
GGA19	Number of SNPs	19755	102	32
	Number of indels	1829	2	2
	Number of genes	84	42	24

With this aim, we used the Variant Effect Predictor (VEP) [23] tool from Ensembl to annotate pre-selected polymorphisms. We therefore focused on “missense”, “stop lost” or “stop gained” annotated coding SNPs, and on “frameshift”, “inframe insertion” and “inframe deletion” annotated coding indels. After a validation procedure of those annotations using VEP on the latest version of the chicken genome, we finally highlighted 120 (including three “stop gained” SNPs and one “frameshift” indel) and 104 (including two “stop gained” SNP, one “frameshift” indels, and one “inframe insertion” indels) candidate polymorphisms occurring in 35 and 42 genes in GGA7 and GGA19 QTL regions, respectively (Table 2).

Considering that important positions in protein and nucleotides sequence have been conserved throughout evolution, we then applied a filter taking conservation into account using NGS-SNP [24]. Indeed, high conservation rate through evolution among different vertebrates may reveal a high selective pressure, and therefore a major impact of substitutions on final protein function. Therefore, selecting SNPs impacting highly conserved regions on orthologous genes may help to focus on potential causal polymorphism underlying QTLs. In this study, we considered two criteria to estimate conservation at a given locus: the first one BLOSUM62-based [24] (SNPs with a |*a*| score >0.27 impact conserved amino-acid residues), and the other one based on SIFT prediction [25] (SNPs annotated as deleterious, see Methods). Considering SNPs that were respectful to either conservation criteria, or to both, the number of functional candidate SNPs was 38 SNPs on GGA7 and to 30 SNPs on GGA19 distributed on 17 and 21 genes, respectively (Table 2, Table S1).

Even if for nonsense SNPs and for indels those criteria cannot be robustly evaluated (see Methods), they were further considered as functional, due to the high impact they may have on final protein products (*i.e.* loss of a part of the protein sequence). Indeed, three indels were identified, located in *PRR11* and two unknown genes (ENSGALG00000021856 and ENSGALG00000005578), with 60%, 12% and 97% of protein loss consequence, respectively. Similarly, three nonsense SNPs out of five were likely to have a drastic effects on the protein structure; they impact *SCN1A*, ENSGALG00000021856 and *OPN1LW* genes, and cause 69%, 41%, and 92% of the proteins loss, respectively (Table 3). None of them have been related to lipid metabolism and therefore represent strong positional but not functional candidate genes for the two QTL regions for adiposity.

**Table 3:** Distribution of functional polymorphisms.

Chromosome	Ensembl gene ID	HGNC	Functional missense SNPs^1^	Nonsense SNPs^2^	Coding indels^3^
GGA7	**ENSGALG00000011149**	**PLA2R1**	7 (12)	-	-
	**ENSGALG00000011153**	**LY75**	3 (5)	-	-
	**ENSGALG00000010858**	**LRP2**	2 (6)	1 (255, 5%)	-
	**ENSGALG00000020703**	**GRB14**	2 (3)	-	-
	**ENSGALG00000010891**	**ABCB11**	1 (2)	-	-
	**ENSGALG00000009545**	**SLC25A12**	1 (1)	-	-
	ENSGALG00000010943	SCN1A	1 (1)	1 (816, 69%)	-
	ENSGALG00000021856	-	-	1 (41, 41%)	1 (12, 12%)
	ENSGALG00000010933	XIRP2	5 (15)	-	-
	ENSGALG00000011068	COBLL1	4 (14)	-	-
	ENSGALG00000011052	SLC38A11	3 (5)	-	-
	ENSGALG00000010956	TTC21B	2 (9)	-	-
	ENSGALG00000014209	GPR155	2 (3)	-	-
	ENSGALG00000013235	PDK1	1 (1)	-	-
	ENSGALG00000009583	GORASP2	1 (2)	-	-
	ENSGALG00000020737	KLHL23	1 (3)	-	-
	ENSGALG00000011110	DPP4	1 (2)	-	-
	ENSGALG00000011172	LOC429030	1 (7)	-	-
GGA19	**ENSGALG00000023554**	**PIGW**	1 (8)	-	-
	**ENSGALG00000005420**	**AATF**	1 (4)	-	-
	**ENSGALG00000005084**	**TRIM37**	1 (2)	-	-
	**ENSGALG00000004917**	**DOC2B**	1 (1)	-	-
	ENSGALG00000005037	TEX14	4 (13)	1 (131, 9%)	-
	ENSGALG00000004924	OPN1LW	-	1 (324, 9%)	-
	ENSGALG00000021526	PRR11	2 (6)	-	1 (176, 60%)
	ENSGALG00000005578	-	-	-	1 (133, 97%)
	ENSGALG00000005061	PPM1E	3 (4)	-	-
	ENSGALG00000005279	BRIP1	2 (2)	-	-
	ENSGALG00000005230	MED13	2 (2)	-	-
	ENSGALG00000005295	BCAS3	2 (2)	-	-
	ENSGALG00000005468	SYNRG	1 (6)	-	-
	ENSGALG00000005350	USP32	1 (5)	-	-
	ENSGALG00000005489	DDX52	1 (4)	-	-
	ENSGALG00000005516	HEATR6	1 (4)	-	-
	ENSGALG00000005173	TUBD1	1 (4)	-	-
	ENSGALG00000005594	OMG	1 (3)	-	-
	ENSGALG00000005269	INTS2	1 (2)	-	-
	ENSGALG00000005285	TBX4	1 (2)	-	-
	ENSGALG00000005362	-	1 (2)	-	-
	ENSGALG00000005868	RAP1GAP2	1 (2)	-	-
	ENSGALG00000011040	SCN2A	1 (2)	-	-
	ENSGALG00000005126	DHX40	1 (1)	-	-

1: Number of SNPs having a potential impact on protein function; Number of total SNPs affecting protein sequence is given in brackets.

2: Number of SNPs having a nonsense impact; Number of amino acids and percentage of protein sequence that are lost are given in brackets.

3: Number of coding indels; Number of amino acids and percentage of protein sequence that are lost are given in brackets.

To sum up, we finally considered 43 and 36 polymorphisms as strong functional candidates, impacting 18 and 23 genes on GGA7 and GGA19 QTL, respectively (Table 2, Table S1 and Table S2). Among those latter one, we selected 3 indels and 8 SNPs to perform Sanger sequencing. All SNP were validated as being polymorphic in our experimental designs. Concerning the 3 indels, this analysis led to the validation of an existing polymorphism in each case, and confirmed the open read frame shift for two of them, while a fine analysis of the one occurring on the chromosome 7 revealed it was not impacting the coding frame as first predicted by VEP and NGS-SNP, but was instead leading to the elongation of the encoded protein. As variants annotation rely on the use of a reference genome, miss-assemblies in such reference could lead to erroneous prediction. Moreover, short indels are usually constituted with homopolymers or tandem repeats, which tends to show higher error rates in re-sequencing data, and negatively impact the mappability of reads supporting them [34]. It appears therefore mandatory to consider carefully indels deeply impacting protein sequences highlighted with high-throughput sequencing approaches and to perform fine validation for further consideration.

### Gene functional information allowed prioritization on genes related to adiposity and associated candidate polymorphisms

Those polymorphisms, selected for being both coding and being functional (*i.e.* having a high propensity for impacting final protein product) were considered as strong candidates underlying QTLs. But, even if the causal polymorphisms may be located in genes that have not already been described as involved in the lipid metabolism, genes known as being related to the trait of interest stand for the first strong candidate genes to be considered. Using gene functional annotations could therefore allow prioritizing polymorphisms among pre-selected candidates. Considering the phenotype targeted in our study, such functional genes were selected using GO and OMIM databases (and genes related to lipid metabolic process, diabetes mellitus and obesity, see Methods). We identified, 6 and 4 functional candidate genes among the 18 and 23 genes with at least one functional polymorphism previously selected on GGA7 and GGA19 QTL regions, respectively. All these genes and associated polymorphisms (17 and 4 for the two regions) are listed in Table S1 and S2. Location and impact of the mutation on the protein and conservation between species are presented in Figure S1.

Among those functional candidates, *SLC25A12* that encodes the Ca^2+^-regulated mitochondrial aspartate-glutamate carrier, is involved in carbohydrates and glucose metabolism and is known as a major autism spectrum disorder susceptibility gene and support oxidative phosphorylation and energy production [35]. *GRB14* and *DOC2B* are involved in regulation of insulin secretion, through insulin receptor binding abilities for the first one [36]–[39] and through insulin vesicle-mediated secretion and uptake capacity for the second one [39]–[42]. *LRP2* encodes for an endocytic receptor known as megalin, which internalizes a variety of ligands such as nutrients, signalling molecules, hormones and lipoproteins and regulates hepatic lipid flow-through [43], [44]. *ABCB11* operate the release of bile salt on the canalicular membrane of hepatocytes and is associated to intrahepatic cholestasis [45], [46]. *PLA2R1* is involved in the modulation of eicosanoid production [47], [48]. *LY75* is involved in antigen presentation and endocytosis, and has been identify as a susceptibility locus for type 2 diabetes mellitus [49], [50]. *TRIM37*, which encodes a peroxysomal protein with E3 ubiquitin ligase known to cause mulibrey nanism when mutated, is also related to severe insulin resistance syndrome [51]. Finally, *PIGW* is involved in the glycosylphosphatidylinositol synthesis [52] and *AATF* in apoptosis inhibition [53].

## Conclusions

Using NGS data allowed the identification of 120 (GGA7) and 104 (GGA19) polymorphisms having an impact on protein sequence. While all of them are good candidates, conservation- and functionality-based filters applied on polymorphisms or genes functional annotation allows us to prioritize a list, with 45 (GGA7) and 36 (GGA19) polymorphisms that might have a strong effect on the protein function (including 7 coding indels or nonsense SNPs), 17 (GGA7) and 4 (GGA19) of them being in functional candidate genes. Even if we cannot exclude that the causal polymorphisms may be located in regulatory regions, this strategy gives a complete overview of the candidate polymorphisms in coding regions, prioritize them and open the way to further validation, by genetic approaches using other populations phenotyped for the traits of interest, or by molecular and cellular functional approaches.

## Supporting Information

Figure S1 **Multi-species protein alignments for a sub-selection of indels and SNPs impacting peptidique sequence.**
(PDF)

Table S1 **Description of functionnal candidate SNPs in the two QTL regions.**
(XLSX)

Table S2 **Description of functionnal candidate indels in the two QTL regions.**
(XLSX)

Table S3 **List of primers used for Sanger sequencing validation.**
(XLSX)
