# Supplementary figures and images for: Re-Sequencing Data for Refining Candidate Genes and Polymorphisms in QTL Regions Affecting Adiposity in Chicken
Source: PLoS One. 2014 Oct 21;9(10):e111299. doi: 10.1371/journal.pone.0111299 (PMC4205046; doi:10.1371/journal.pone.0111299)

Nonsens SNPs

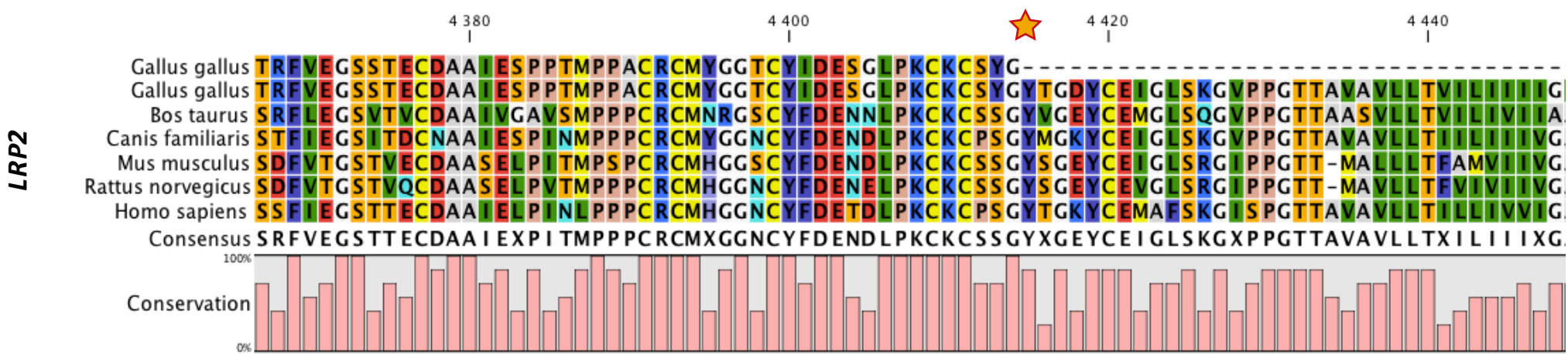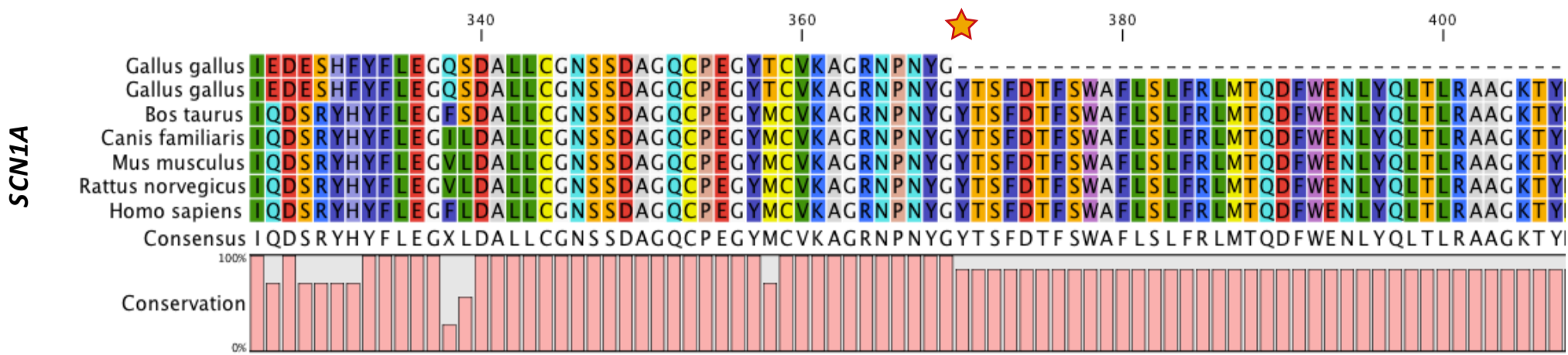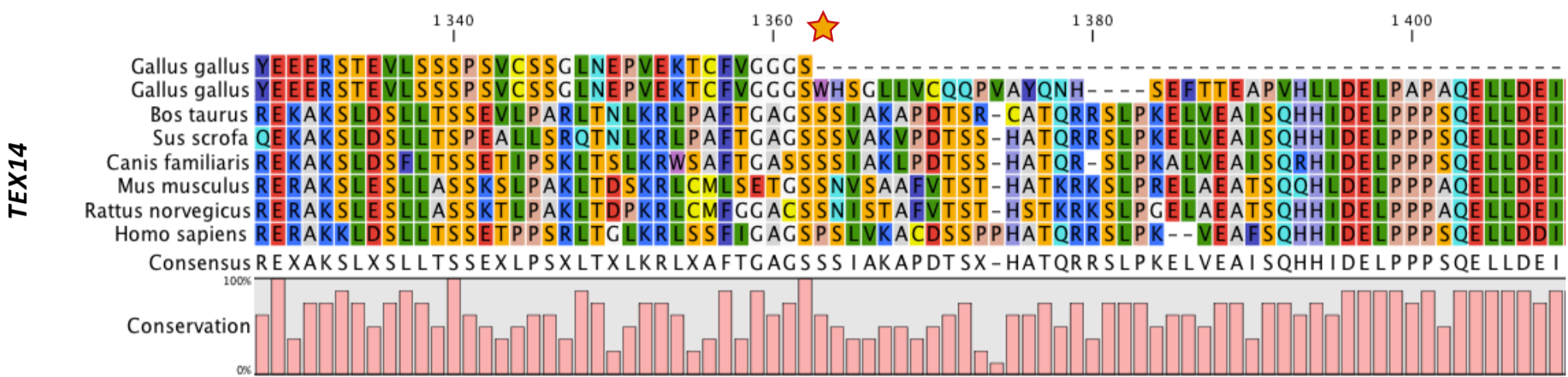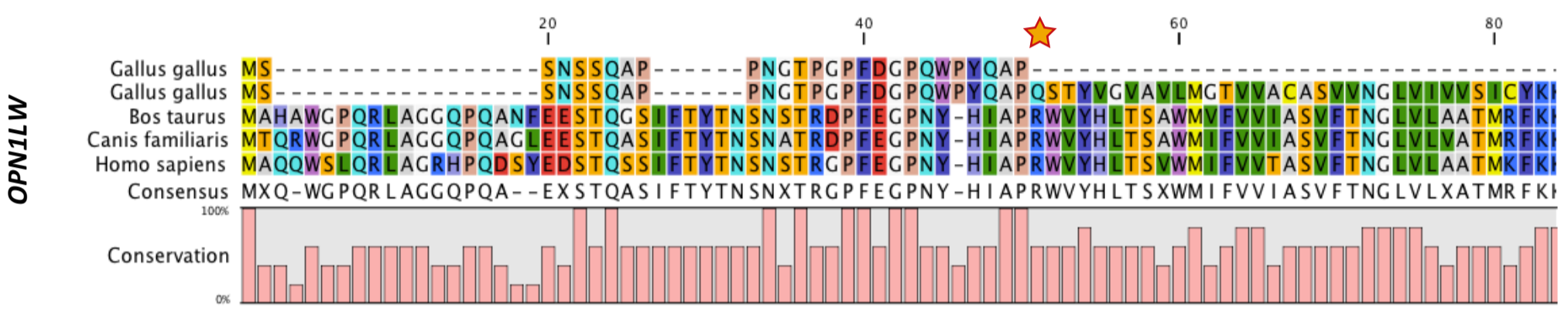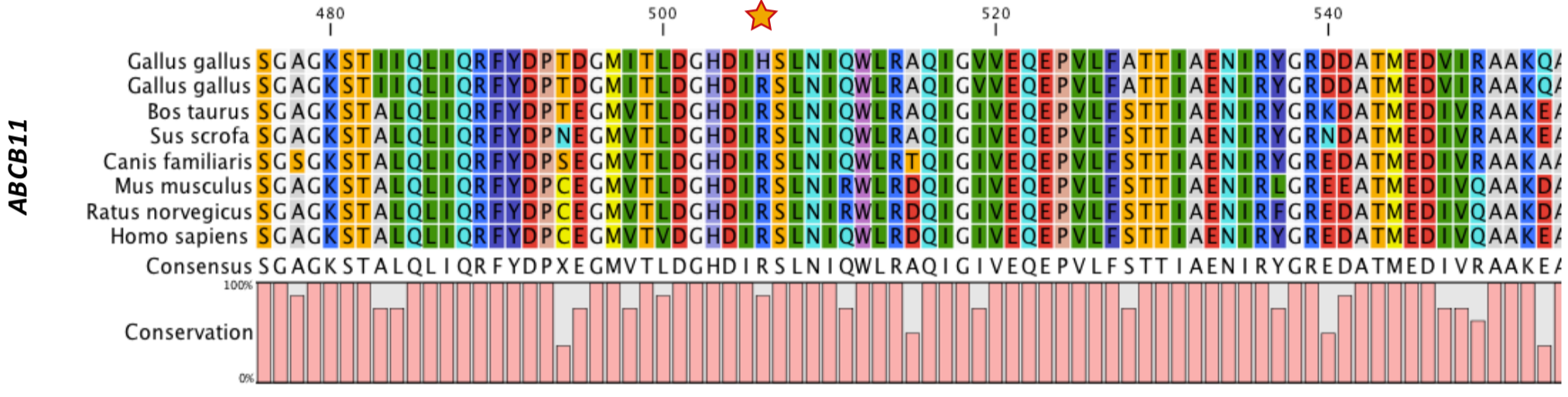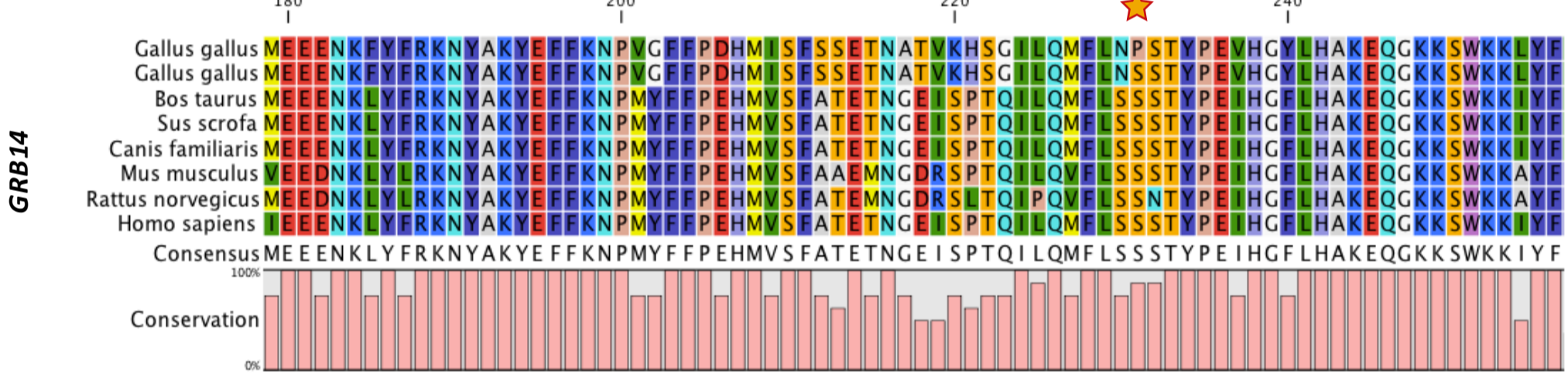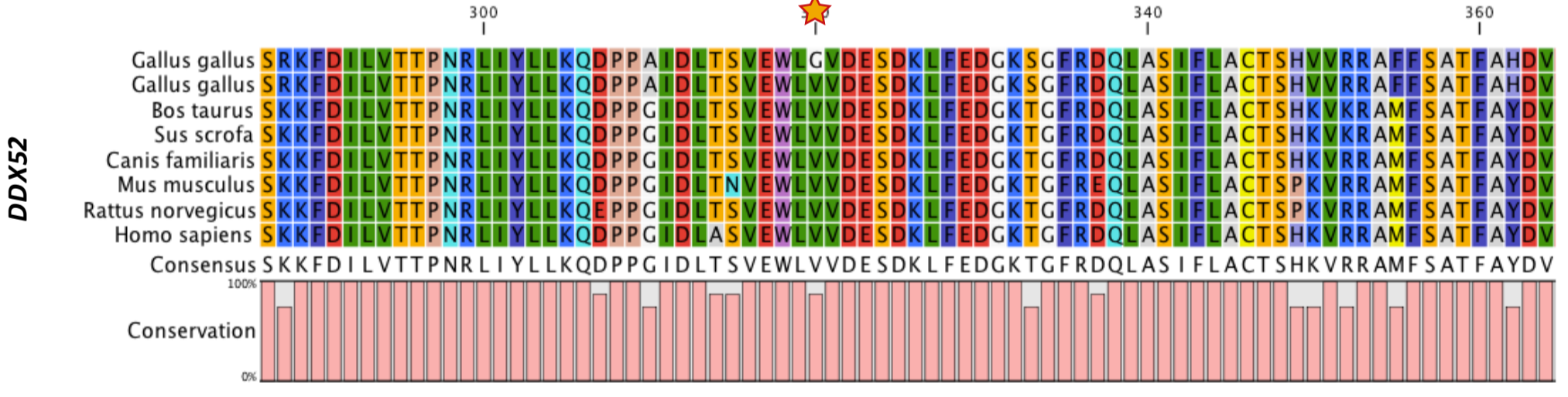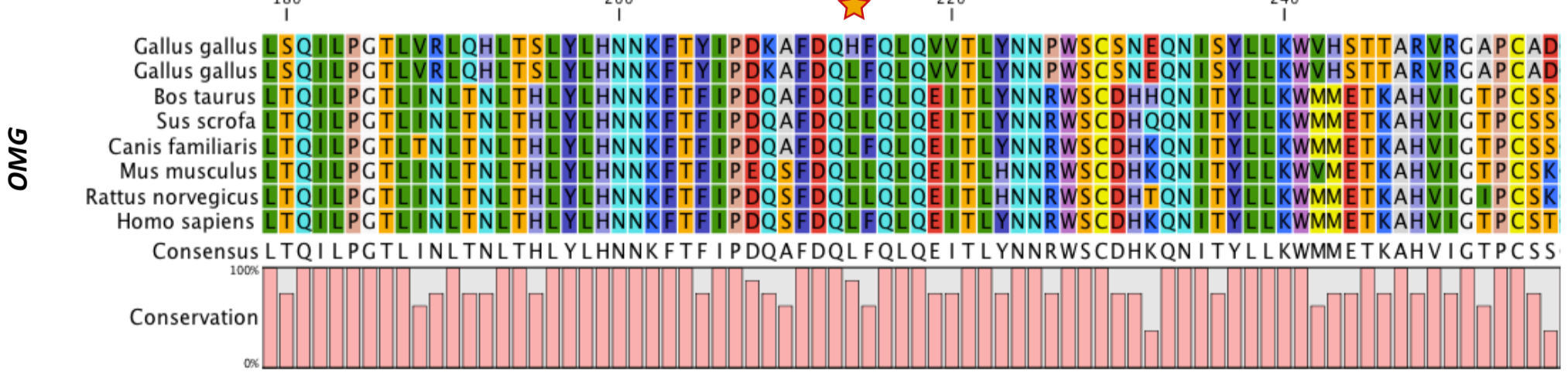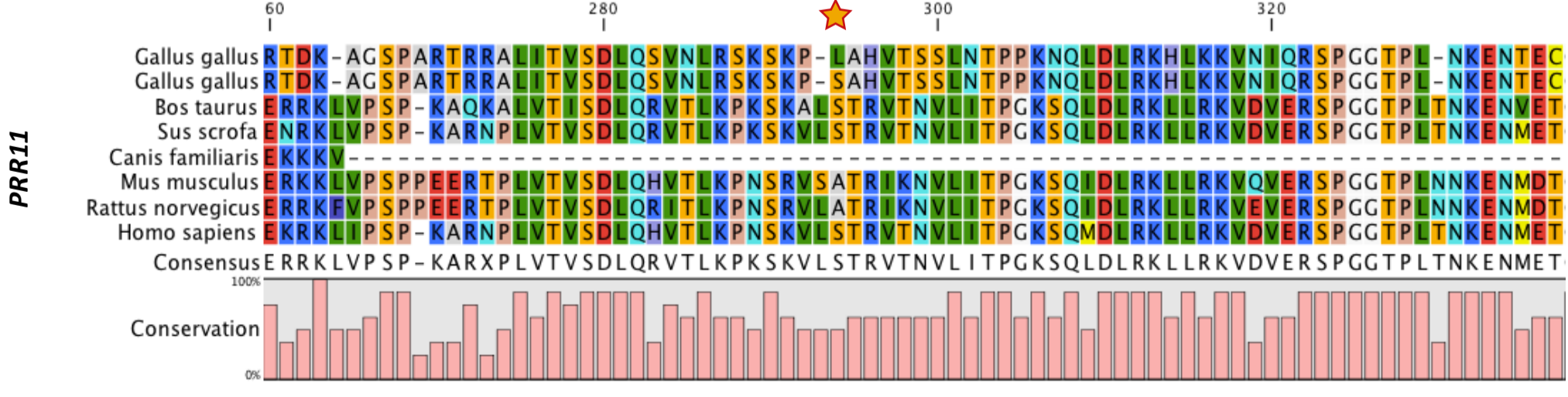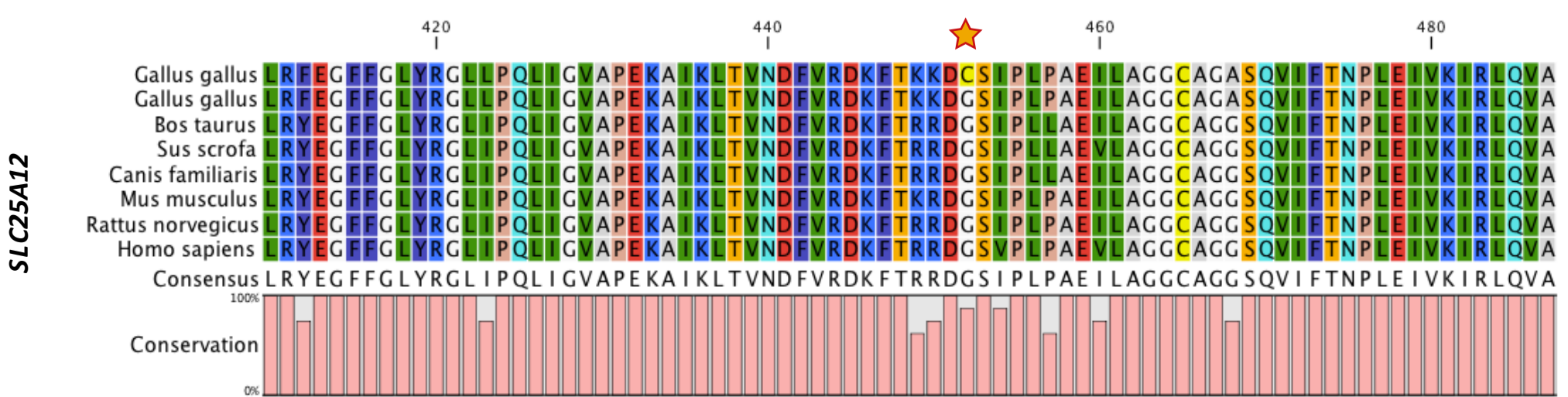

Supplement: Figure S1 — Multi-species protein alignments for a sub-selection of indels and SNPs impacting peptidique sequence. (PDF) [file pone.0111299.s001.pdf]
